# Competitive Fitness of Influenza B Viruses Possessing E119A and H274Y Neuraminidase Inhibitor Resistance–Associated Substitutions in Ferrets

**DOI:** 10.1371/journal.pone.0159847

**Published:** 2016-07-28

**Authors:** Philippe Noriel Q. Pascua, Bindumadhav M. Marathe, Andrew J. Burnham, Peter Vogel, Richard J. Webby, Robert G. Webster, Elena A. Govorkova

**Affiliations:** 1 Department of Infectious Diseases, St. Jude Children’s Research Hospital, Memphis, Tennessee, United States of America; 2 Gryphon Scientific, Takoma Park, Maryland, United States of America; 3 Veterinary Pathology Core, St. Jude Children’s Research Hospital, Memphis, Tennessee, United States of America; University of Geneva, SWITZERLAND

## Abstract

Neuraminidase (NA) inhibitors (NAIs) are the only antiviral drugs recommended for influenza treatment and prophylaxis. Although NAI-resistant influenza B viruses that could pose a threat to public health have been reported in the field, their fitness is poorly understood. We evaluated in ferrets the pathogenicity and relative fitness of reverse genetics (rg)–generated influenza B/Yamanashi/166/1998-like viruses containing E119A or H274Y NA substitutions (N2 numbering). Ferrets inoculated with NAI-susceptible rg–wild-type (rg-WT) or NAI-resistant (rg-E119A or rg-H274Y) viruses developed mild infections. Growth of rg-E119A virus in the nasal cavities was delayed, but the high titers at 3 days post-inoculation (dpi) were comparable to those of the rg-WT and rg-H274Y viruses (3.6–4.1 log_10_TCID_50_/mL). No virus persisted beyond 5 dpi and replication did not extend to the trachea or lungs. Positive virus antigen-staining of the nasal turbinate epithelium was intermittent with the rg-WT and rg-H274Y viruses; whereas antigen-staining for the rg-E119A virus was more diffuse. Virus populations in ferrets coinoculated with NAI-susceptible and -resistant viruses (1:1 mixture) remained heterogeneous at 5 dpi but were predominantly rg-WT (>70%). Although the E119A substitution was associated with delayed replication in ferrets, the H274Y substitution did not measurably affect viral growth properties. These data suggest that rg-H274Y has undiminished fitness in single virus inoculations, but neither rg-E119A nor rg-H274Y gained a fitness advantage over rg-WT in direct competition experiments without antiviral drug pressure. Taken together, our data suggest the following order of relative fitness in a ferret animal model: rg-WT > rg-H274Y > rg-E119A.

## Introduction

Influenza is an acute respiratory viral infection that causes annual global epidemics resulting in significant morbidity and mortality in humans. Although these epidemics are caused by both influenza A and B viruses, members of the *Orthomyxoviridae* family of single-stranded RNA viruses, the disease burden caused by influenza B viruses has been greatly overshadowed by that caused by influenza A viruses. However, recent surveillance and epidemiologic data suggest that, in some influenza seasons, the consequences of influenza B virus infections, including the clinical disease severity and the inflammatory response, are almost equivalent to those of influenza A virus infections [1–4]. In some severe cases of human infection, particularly among children, influenza B virus has established a lower respiratory tract (LRT) infection and induced acute respiratory distress syndrome, as well as influenza-associated myositis and gastroenteritis [1,5,6]. Data from the Centers for Disease Control and Prevention indicate that, from 2010 to 2015, an average of 47.6% (range, 22.6% to 84.6%) of tallied influenza cases in the United States were caused by influenza B virus infections, accounting for an average of 41.1% (range, 15.3% to 76.8%) of influenza-associated pediatric deaths in each influenza season [7,8]. Based on available data, the average global percentage of circulating influenza B viruses since 2003 remains relatively low at 21–22% compared to that of influenza A viruses [9].

Two antigenically distinct lineages of influenza B viruses (i.e., Victoria and Yamagata, named after their progenitor strains) co-circulate globally and tend to cycle in frequency (i.e., they predominate in some influenza seasons while being less prevalent in other years). Vaccination remains the primary measure for controlling influenza disease, and since 2012 representative strains of both lineages of influenza B virus have been included in FDA-approved quadrivalent seasonal influenza vaccines [10,11]. In the absence of available vaccines, antiviral treatment is an effective alternative option for controlling influenza. Neuraminidase (NA) inhibitors (NAIs) target the NA surface glycoproteins of influenza A and B viruses. Through competitive binding to the active site of NA, NAIs inhibit NA-mediated cleavage of virus-associated sialic acids (SA) expressed on epithelial cell surface during virus budding, thereby preventing the release and spread of newly formed infectious progeny virions. NAIs are currently the only class of antivirals recommended for treatment and prophylaxis of influenza B virus infections worldwide. Thus, the emergence and spread of NAI-resistant influenza B viruses would present a public health concern.

NAI-resistant influenza A and B viruses can emerge under drug selection pressure or appear naturally without drug intervention through associated amino acid substitutions, typically at one of 19 highly conserved residues in or near the NA active site [12,13]. Influenza B viruses associated with reduced susceptibility to NAIs as a result of amino acid substitutions at positions 105, 110, 119, 152, 198, 222, 250, 274, 294, 371, or 402 (N2 numbering) have been detected in surveillance studies or isolated from patients undergoing NAI treatments [9,14]. NAI resistance mutations may also have differing effects on virus fitness, defined as the summation of all parameters that quantify the degree of virus adaptation in a given environment or host [15], producing variants that may have diminished, undiminished, or superior fitness compared to their wild-type (WT) virus counterparts [15,16]. However, in contrast to influenza A viruses, for which the effect of some of these amino acid substitutions on NA function and viral fitness has been well characterized [13], corresponding data and information on influenza B viruses are limited and are mostly derived from *in vitro* studies. In cell culture experiments, recombinant B/Beijing/1/1987 viruses (Victoria lineage) with the E119D, R152K, or R292K NA substitution demonstrated significantly impaired growth in MDCK cells, whereas possession of the E119A/V/G substitution induced only minor or no impairment of the replication kinetics [17]. Similarly, introducing the D198T, D198E, or R371K NA substitution in B/Yamanashi/166/1998 (Yamagata lineage) virus restricted its replication efficiency *in vitro*, but E119A, I222T, H274Y, or N294S substitutions had no such effect [18].

Ferrets have been extensively used to model human infections with influenza A viruses, including the seasonal A(H1N1) and A(H3N2), highly pathogenic A(H5N1), and 2009 pandemic H1N1 [A(H1N1)pdm09] viruses, because of their natural susceptibility to infection and their manifestation of clinical respiratory disease and lung pathology similar to those seen in humans [19–21]. Influenza B virus infection in ferrets is generally mild [22–24], but certain strains may cause severe clinical disease [25]. Upon inoculation with influenza B viruses, ferrets also demonstrate influenza-like disease and immune responses comparable to those in humans, indicating their suitability as an animal model for assessing disease outcome and virus pathogenicity [25,26]. However, the effect of NAI resistance–associated substitutions on the fitness of NAI-resistant influenza B viruses, as compared with that of their drug-susceptible counterparts in animal models, remains largely unknown. To date, only two independent studies have examined the effect of catalytic R152K or framework D198N NA substitution on the fitness of influenza B viruses in competitive coinoculation ferret experiments [27,28].

To study the effect of NAI resistance–associated E119A and H274Y framework NA substitutions in the homogeneous background of the B/Yamanashi/166/1998 virus (Yamagata lineage) in a ferret animal model, we examined (1) traditional virus-host interactions and (2) virus-virus interaction within a host (competitive fitness). These two NA substitutions are naturally occurring and were previously identified in clinical and surveillance studies [9,14]. In *in vitro* studies, the E119A NA substitution resulted in highly reduced inhibition by three NAIs (oseltamivir, zanamivir, peramivir), whereas H274Y promoted reduced inhibition by oseltamivir and peramivir [18]. The acquisition of H274Y afforded a fitness advantage over parental WT virus in a competitive coinfection cell culture environment and thus did not impair viral fitness [29]. Additionally, H274Y and E119A are the most commonly reported NAI resistance–associated NA substitutions for the N1 and N2 subtypes of influenza A viruses, respectively [14].

## Materials and Methods

### Ethics statement

Protocols and procedures followed throughout the study were approved by the St. Jude Animal Care and Use Committee and complied with the policies of the National Institutes of Health and the Animal Welfare Act. All animal experiments were conducted at St. Jude Children's Research Hospital (Memphis, Tennessee, United States of America) under applicable laws and guidelines and after approval from the IACUC.

### Cells and viruses

Madin-Darby canine kidney (MDCK) cells were obtained from the American Type Culture Collection (Manassas, VA) and maintained in Eagle’s minimal essential medium (EMEM; Life Technologies, Grand Island, NY) containing 5% heat-inactivated fetal bovine serum (GE; Logan, UT) supplemented with 2 mM L-glutamine, 0.2% sodium bicarbonate, vitamin solution, antibiotic-antimycotic solution, and 40 μg/mL gentamicin (Life Technologies).

Recombinant WT B/Yamanashi/166/1998 (Yamagata lineage) virus (rg-WT) or influenza B viruses containing a single NA substitution (rg-E119A or rg-H274Y) were generated and rescued by the reverse-genetics (rg) method using pAD3000 plasmid vectors as previously described [29,30] in accordance with experimental protocols approved by the Institutional Biosafety Committee of St. Jude Children’s Research Hospital. NA sequences were confirmed and virus stocks were propagated and prepared by infecting MDCK cells at 33°C for 72 to 96 h, and stored at −80°C until use.

### Assessment of influenza B virus pathogenicity in ferrets

Young adult male ferrets aged 4 to 5 months were purchased from Triple F Farms (Sayre, PA). All ferrets were seronegative by hemagglutination inhibition (HI) testing for human seasonal influenza A(H1N1) and A(H3N2) and influenza B (Victoria and Yamagata lineages) viruses. Groups of ferrets were lightly anesthetized with isoflurane and inoculated intranasally with 10^6^ plaque-forming units (PFU) in 1 mL phosphate-buffered saline (PBS) (administered as 500 μL per nostril) of either homogenous populations of each virus (rg-WT, rg-E119A, or rgH274Y) or 1:1 mixtures of NAI-susceptible rg-WT and -resistant rg-E119A or rgH274Y virus pairs at equivalent ratios (5 × 10^5^ PFU and 5 × 10^5^ PFU, respectively) in 1 mL PBS. Clinical signs of infection (weight loss, temperature increase, change in activity score [31], and respiratory disease indications, such as sneezing, wheezing, nasal discharge or exudate, and congestion) were monitored daily (*n* = 4/group) up to 14 days post-inoculation (dpi). Body weight changes were measured relative to starting weight (day 0), whereas temperature elevations were recorded once daily by using subcutaneous implantable temperature transponders (Bio Medic Data Systems Inc., Seaford, DE).

Ferrets (*n* = 4/group) were anesthetized by intramuscular injection of ketamine HCl (25 mg/kg of body weight), and virus shedding in the upper respiratory tract (URT) was monitored at 1, 3, 5, and 7 dpi by instilling 0.5 mL sterile PBS into each nostril and collecting nasal washes. Separately, ferrets (*n* = 3/group) were euthanized by intracardiac injection of barbiturate overdose (0.5 mL) under deep isoflurane anesthesia at 3 dpi, and tissue samples (0.5 g) were collected from their trachea and lungs (the right cranial and left caudal lobes being collected and processed separately). For virus titration, samples were homogenized in 1 mL sterile PBS with antibiotics. All animal experiments were conducted under the applicable laws and guidelines and were approved by the Animal Care and Use Committee of St. Jude Children’s Research Hospital.

### Histopathologic examination and immunohistochemical (IHC) staining

Nasal turbinates and lung tissues collected at 3 dpi were perfused with 10% neutral buffered formalin (NBF, Thermo Scientific) at necropsy. Excised tissues were further preserved in 10% NBF for at least 7 days before paraffin embedding, sectioning, and staining. Sections of each tissue underwent standard hematoxylin and eosin staining and were examined under light microscopy. IHC staining was performed using goat antiserum raised against the HA glycoprotein of B/Florida/04/2006 (Yamagata lineage) virus. Specific antigen–antibody reactions were visualized by staining with 3,3,9-diaminobenzidine tetrahydrochloride (Vector Laboratories, Inc.; Burlingame, CA).

### Virus infectivity titrations

Influenza B virus infectivity in virus stocks, ferret nasal washes, and supernatants from homogenized tissue samples was determined in MDCK cells by 50% tissue culture infectious dose (TCID_50_) assays after 72 to 96 h incubation at 33°C. Confluent monolayers of MDCK cells in 96-well plates were inoculated with 10-fold serial dilutions of each sample in quadruplicate in the presence of 1 μg/mL L-tosylamido-2-phenylmethyl chloromethyl ketone (TPCK)–treated trypsin (Worthington, Lakewood, NJ). Virus replication was detected in supernatants by a hemagglutinin (HA) assay with 0.5% chicken red blood cells (cRBCs), and virus titers were determined by the endpoint method [32]. Viral titers were expressed as log_10_ TCID_50_ per milliliter (mL) or per gram (g), with the limit of detection being set at less than 0.75 log_10_ TCID_50_/mL or TCID_50_/g.

### Laboratory indicators of inflammation in the URT of ferrets

Inflammatory cell counts were determined in the nasal washes of inoculated ferrets (*n* = 4/group) collected at 1, 3, 5, and 7 dpi. Briefly, nasal washes from individual ferrets were centrifuged at 2000 rpm for 10 min, the neat pellet was resuspended in cold PBS, and the cells were counted using a FORCYTE hematology analyzer (Oxford Science, Oxford, CT). Absolute cell numbers from individual ferrets were used for calculation and comparison of values obtained between experimentally inoculated groups. Mock-inoculated control ferrets (*n* = 4/group) were inoculated intranasally with 1 mL PBS (administered as 500 μL per nostril), a volume equivalent to the virus inoculum used.

### Serologic tests

Serum samples were collected from individual ferrets 3 weeks after virus inoculation, treated with receptor-destroying enzyme (Denka Seiken Co., Ltd., Tokyo, Japan), heat-inactivated at 56°C for 30 min, and tested by HI assay with 0.5% packed cRBCs.

### Clonal sequence analysis

Viral RNA was isolated directly from nasal washes by using an RNA isolation kit (RNeasy, Qiagen, Valencia, CA). NA gene segments were amplified using gene-specific primers [29] and Platinum *Taq* DNA polymerase (Life Technologies) with a SuperScript III One-Step RT-PCR System. PCR amplicons were gel extracted with a QIAquick Gel Extraction Kit (Qiagen), cloned into a pCR 2.1 TOPO TA cloning vector (Life Technologies), and transformed with chemically competent Top10 One Shot *E*. *coli* cells. Individual colonies carrying the gene of interest were randomly picked and subjected to sequence analysis. Plasmid DNA was extracted from bacterial cells grown overnight by using a QIAprep Spin Miniprep Kit (Qiagen) and sequenced with NA gene-specific primers (B/NA-F2: 5’-GCACTCCTAATTAGCCCTCATAGA-3’ paired with B/Yamanashi-NA-441R: 5’- CATTGTAGTATCCCCCTGGTTGG– 3’ or B/NA-1487R: 5’-TAAGGACAATTGTTCAAAC-3’). The DNA template was sequenced using BigDye^®^ Terminator v3.1 Cycle Sequencing Kit by the Hartwell Center for Bioinformatics and Biotechnology at St. Jude Children’s Research Hospital. Samples were analyzed with a Perkin-Elmer Applied Biosystems DNA sequencer (model 3730xl). DNA sequences were completed and edited using the Lasergene sequence analysis software package (DNASTAR, Madison, WI).

### RT-PCR using influenza B virus matrix (M) gene–specific primers

Virus replication in tissue samples was additionally confirmed by RT-PCR targeting the viral M gene sequence. Briefly, viral RNA was isolated directly from supernatants of tracheal and lung tissue homogenates (*n* = 3/group) by using an RNA isolation kit (RNeasy, Qiagen). M gene segments were amplified using gene-specific primers (BM-1F: 5’-AGCAGAAGCAGGCACTTTCT-3’ and B/M-1187R: 5’-TAGAAACAACGCACTTTTTC-3’) and Platinum *Taq* DNA polymerase (Life Technologies) with a SuperScript III One-Step RT-PCR System. PCR amplicons were visualized by agarose gel electrophoresis.

### Statistical analysis

Virus titers and cell counts in nasal wash samples and changes in ferrets’ temperature and weight were compared in one-way analysis of variance (ANOVA) with Bonferroni’s multiple comparison post-test (GraphPad Prism 5.0 software). The proportions of the rg-WT and the NAI-resistant viruses in the coinoculation experiments were also tested in repeated measures ANOVA.

## Results

### Clinical features of infection with NAI-susceptible and -resistant influenza B viruses in ferrets

To evaluate the effect of a single NA amino acid substitution on the fitness of B/Yamanashi/166/1998-like viruses in a ferret model, we compared the disease progression (in terms of weight loss, temperature elevation, and activity score) and clinical signs of respiratory disease (sneezing and nasal discharge) induced by the parental rg-WT virus and the NAI-resistant rg-E119A and rg-H274Y viruses. Consistent with previous reports [22–25], inoculating ferrets with the recombinant rg-WT virus induced mild disease with no significant weight loss or increase in body temperature relative to the pre-inoculation baseline measurements. A transient elevation of body temperature was detected at 2 dpi, with a maximum average change of 0.7 ± 0.5°C (Table 1). Comparable mean temperature changes were also displayed by ferrets inoculated with the rg-H274Y (0.7 ± 0.1°C) and rg-E119A (0.7 ± 0.3°C) viruses at 2 and 3 dpi, respectively. One ferret from the rg-E119A virus infection group lost 3.5% of its body weight at 5 dpi but regained it soon after. The activity levels of all groups were unaffected, and nasal discharge, including sneezing, was not prominent.

**Table 1:** Clinical features of infections and replication kinetics of NAI-susceptible and -resistant influenza B viruses in the URT of ferrets.

Recombinant B/Yamanashi/166/1998 influenza virus	No. of ferrets showing indicated clinical signs of disease/total no.			Nasal wash virus titers (mean ± SD, log_10_TCID_50_/g) on day postinoculation with virus (no. positive/total no.)^b^			
	Weight loss (mean ± SD, %)	Temp increase (mean ± SD,°C)	Respiratory^a^	1 dpi	3 dpi	5 dpi	7 dpi
Virus-host interaction							
rg-WT	0/4	4/4 (0.7 ± 0.5)	0/4	3.6 ± 0.1 (4/4)	3.6 ± 0.44 (3/4)	3.0 ± 0.3 (2/4)	< (0/4)
rg-E119A	0/4	4/4 (0.7 ± 0.3)	0/4	0.750 ± 0.0 (4/4)^c^^,^^d^	4.1 ± 0.3 (4/4)	2.2 ± 0.1 (3/4)	< (0/4)
rg-H274Y	1/4 (3.5)	4/4 (0.7 ± 0.1)	0/4	3.1 ± 0.5 (4/4)	3.9 ± 0.4 (2/4)	3.0 ± 0.4 (4/4)	< (0/4)
Virus-virus interaction within the host							
rg-WT:rg-E119A	3/4 (2.1 ± 0.6)	4/4 (0.5 ± 0.1)	0/4	2.8 ± 0.3 (4/4)^e^	3.9 ± 0.4 (4/4)	2.8 ± 0.3 (3/4)	< (0/4)
rg-WT:rg-H274Y	0/4	4/4 (1.0 ± 4)	0/4	2.4 ± 0.1 (3/4)^e^	3.7 ± 0.1 (3/4)	2.9 ± 0.2 (4/4)	< (0/4)

Groups of four ferrets were inoculated with 10^6^ PFU in 1 mL PBS of recombinant B/Yamanashi/166/1998-derived rg-WT, rg-E119A, or rg-H274Y virus. Two additional groups of ferrets were coinoculated with a mixture (1:1 ratio) of the NAI-susceptible rg-WT (5 × 10^5^ PFU) and NAI-resistant virus (rg-E119A or rg-H274Y, at 5 × 10^5^ PFU) in 1 mL PBS. All inoculated ferrets seroconverted (320–640 reciprocal titers of anti-HA antibodies) against homologous virus at 21 dpi.

^a^Respiratory signs monitored include sneezing and nasal discharge.

^b^Expressed as log_10_TCID_50_/mL. <, below limit of virus detection (0.75 log_10_TCID_50_/mL).

^c^*P* < 0.001, relative to rg-WT values.

^d^*P* < 0.05, relative to rg-H274Y values.

^e^*P* < 0.05, relative to rg-WT values.

We also evaluated the effect of the presence of equal proportions of NAI-susceptible and -resistant viruses in the virus inoculum on the magnitude and duration of clinical signs of infection. Separate groups of ferrets (*n* = 4/group) were coinoculated intranasally with a mixture of rg-WT and one of the NAI-resistant viruses (rg-E119A or rg-H274Y) at a 1:1 ratio. As in the groups inoculated with homogenous virus population, mild disease progression was observed in the coinoculation groups. Three of the four ferrets inoculated with the rg-WT:rg-E119A virus mixture experienced mild weight loss (2.1 ± 0.6%) at 2 to 3 dpi (Table 1). The body temperatures of two ferrets barely increased (0.2 ± 0.1°C) at 2 dpi, whereas the remaining two ferrets experienced a maximum mean increase of 0.9 ± 0.8°C at 5 dpi. In contrast, an average increase of 1.0 ± 0.4°C in body temperature was recorded in the four ferrets that received rg-WT:rg-H274Y virus mixture, but none of these animals exhibited weight loss (Table 1). No changes in activity level or clinical signs of respiratory disease were evident in any of these coinoculation groups. Therefore, there were no distinct differences in the clinical signs or duration of influenza-like illness in ferrets inoculated with NAI-susceptible (rg-WT) or -resistant viruses (rg-E119A or rg-H274Y) or in animals coinoculated with a mixture of viruses (rg-WT: rg-E119A or rg-WT:rg-H274Y).

**Fig 1:**
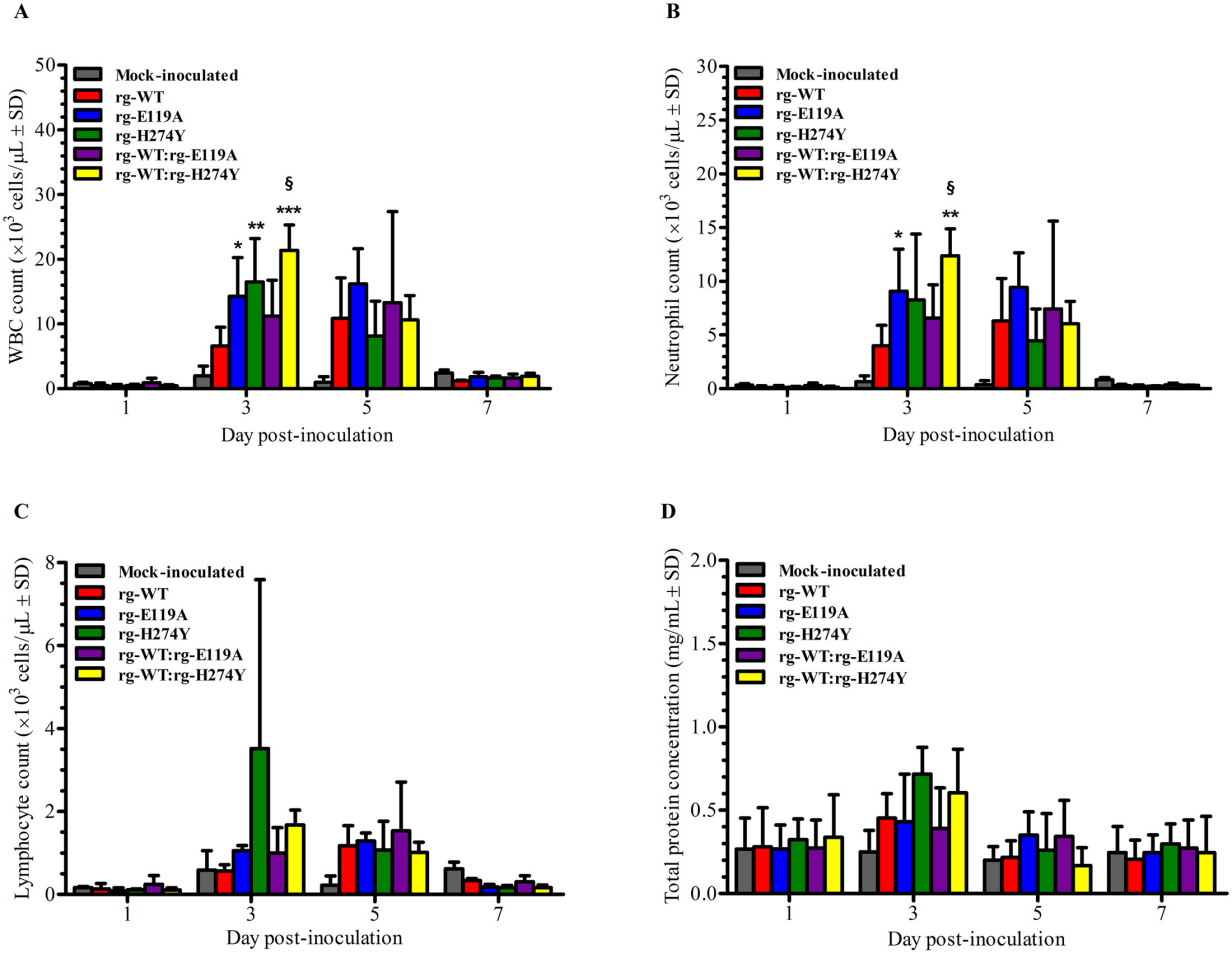
Laboratory indicators of inflammation in nasal washes of ferrets inoculated with NAI-susceptible or–resistant influenza B viruses. Ferrets (*n* = 4/group) were inoculated intranasally with 10^6^ PFU in 1 mL PBS of rg-WT, rg-E119A, rg-H274Y, or a mixture (1:1 ratio) of the NAI-susceptible rg-WT (5 × 10^5^ PFU) and either of the NAI-resistant (rg-E119A or rg-H274Y) (5 × 10^5^ PFU) influenza B virus. Mock-inoculated control ferrets (*n* = 4/group) were inoculated intranasally with PBS. Inflammatory cell counts in the URT were determined in the nasal washes of inoculated ferrets at 1, 3, 5, and 7 dpi. Cell counts were based on the differences in relative size (impedance) and complexity (light scatter) of cells in the sample. Bars represent mean values ± SD. Statistically significant differences relative to mock-inoculated control (*, *P* < 0.05; **, *P* < 0.01; ***, *P* < 0.001) or rg-WT-inoculated ferrets (§, *P <* 0.05) were analyzed by one-way ANOVA with Bonferroni’s multiple comparison post-test.

Inflammation and cell recruitment relies on the presence of replicating influenza virus [24]. Thus, laboratory indicators of inflammation at the nasal cavity of ferrets were also investigated as potential parameter for influenza B virus pathogenicity as previously done with influenza A viruses [33–38]. For these, the elevations of white blood cells (WBCs), neutrophils, lymphocytes, and total protein counts in nasal washes were examined. We recorded some degree of changes in cellularity in the URT of virus-inoculated ferrets particularly at 3 and 5 dpi relative to the mock-inoculated control group. This finding clearly indicates that virus inoculation but not the saline solution inoculation (PBS) was responsible for the observed changes in cellularity in the URT of ferrets (Fig 1). Compared to the mock-inoculated control group, significant elevation of WBC and neutrophil cell counts were afforded by inoculation with rg-E119A (*P* < 0.05 and *P* < 0.01, respectively) and rg-H274Y viruses (*P* < 0.01, WBC only), and a mixture of rg-WT:rg-H274Y viruses (*P* < 0.001 and *P* < 0.01, respectively). Although most of the virus-inoculated groups had appreciably higher counts than the rg-WT group at 3 and 5 dpi (Fig 1A–1C), only the rg-WT:rg-H274Y coinoculation group demonstrated significantly higher WBCs (*P* < 0.01) and neutrophils (*P* < 0.05) in the nasal washes relative to those in the rg-WT group at 3 dpi. Despite such elevations, these induction levels remained fairly low, confirming previous observations that infection with influenza B viruses in ferrets only causes weak inflammatory responses [22]. Cell counts had subsided to normal levels in all groups by 7 dpi.

### Replication fitness of NAI-susceptible and -resistant influenza B viruses in the URT and LRT of ferrets

Influenza A virus infection in ferrets is primarily a URT infection, and serial sampling of nasal washes allows the recovery and titration of infectious viruses shed in the nasal secretions [31]. Previous studies have also shown that influenza B viruses can be shed by ferrets in the URT [23–25,27,28]. The parental rg-WT virus successfully replicated at 1 dpi, producing a slightly higher but not significantly different viral titer relative to the rg-H274Y virus (mean, 3.6 versus 3.9 log_10_TCID_50_/mL) (Table 1). In contrast, the rg-E119A virus did not induce detectable nasal wash titers at this time-point (*P* < 0.001, relative to rg-WT and rg-H274Y) (Table 1). However, it eventually reached a high mean viral titer at 3 dpi, which was comparable to those of the rg-WT and rg-H274Y viruses (range, 3.6 to 4.1 log_10_TCID_50_/mL; *P* > 0.05). Individual viruses sustained growth up to 5 dpi, with rg-E119A producing the lowest nasal wash titers (8-fold lower than those obtained with rg-WT) at this time-point; no infectious virus was recovered from any of the nasal washes collected at 7 dpi (Table 1).

In ferrets coinoculated with the rg-WT:rg-E119A or rg-WT:rg-H274Y virus mixtures, nasal wash titers were significantly lower (2.8 and 2.4 log_10_TCID_50_/mL, respectively) than in the parental rg-WT–inoculated group (*P* < 0.05) at 1 dpi (Table 1). However, there were no significant differences between the nasal wash viral titers in the coinoculated ferrets and those in animals inoculated with a single virus at 3 dpi and production of infectious viruses were not sustained beyond 5 dpi. Thus, virus-virus interaction within a host reflected a trend in replication kinetics comparable to that observed in ferret groups inoculated with a single virus.

To determine whether WT and NAI-resistant influenza B viruses were able to replicate in the LRT of ferrets, we aimed to detect viruses in the trachea and two lung lobes (*n* = 3/group). However, no infectious virus was recovered from any of the tracheal and lung tissues collected from ferrets at 3 dpi. The apparent absence of virus replication was confirmed by the negative results of RT-PCR assays specifically targeting the viral M gene. All the ferrets seroconverted at 21 dpi, with reciprocal geometric mean HI titers of 320 to 640 that were comparable across the inoculation groups. Overall, these results revealed that the replication of both the NAI-susceptible and -resistant B/Yamanashi/166/1998-derived viruses is limited to the URT of ferrets and that the viruses are unable to establish productive replication in lung tissues. More importantly, however, the rg-H274 virus, but not the rg-E119A variant, replicated without apparent replication fitness cost in this mammalian model.

### Immunohistopathologic changes associated with NAI-susceptible and -resistant influenza B viruses

**Fig 2:**
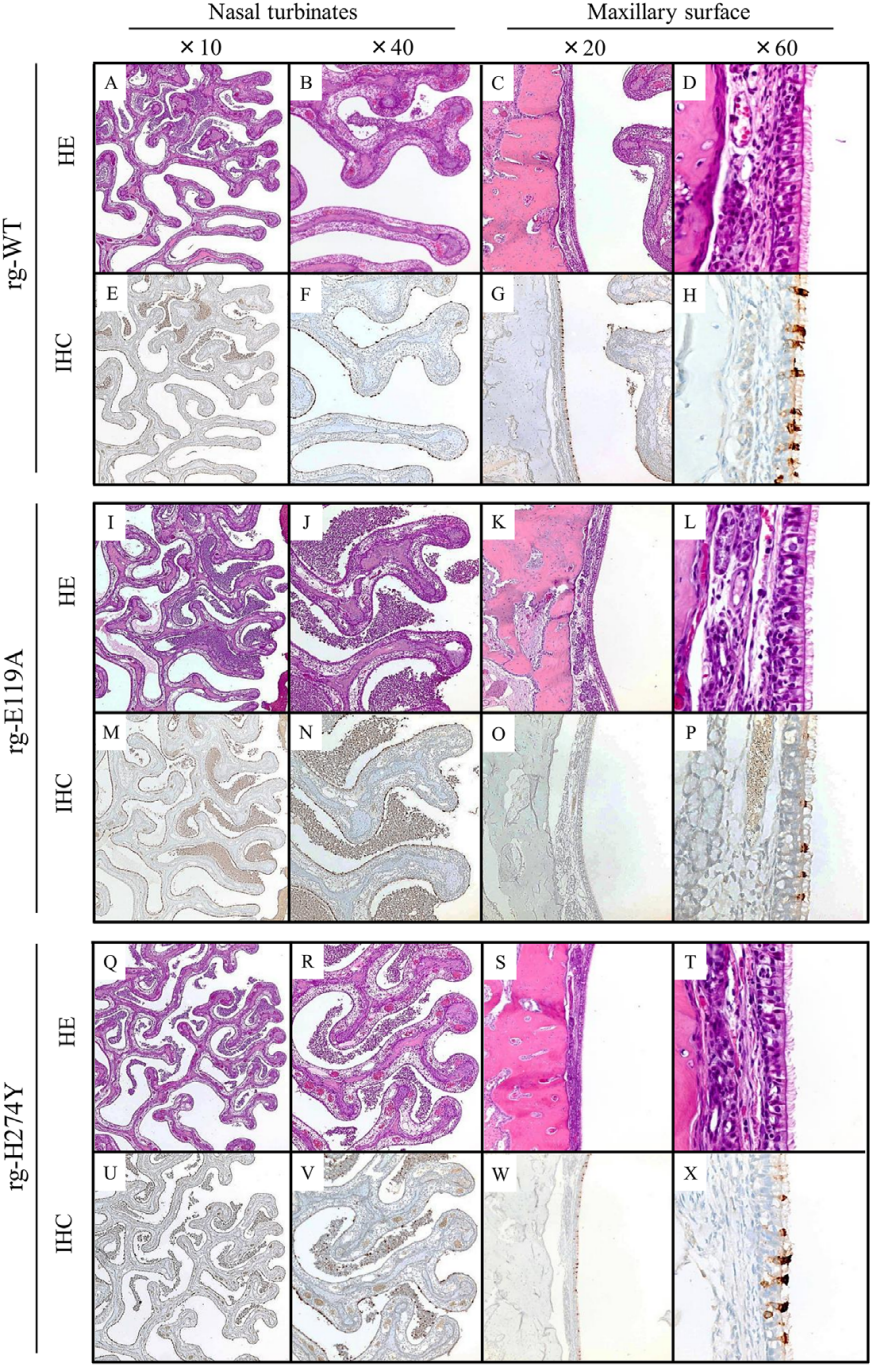
Immunohistopathology of respiratory tract tissues of ferrets inoculated with NAI-susceptible or–resistant influenza B viruses. Ferrets (*n* = 3/group) were inoculated intranasally with 10^6^ PFU in 1mL PBS of rg-WT, rg-E119A, or rg-H274Y influenza B virus. Respiratory tract tissues, including nasal turbinates, trachea, and lungs lobes (cranial and caudal), were collected at 3 dpi for histopathologic examination. Tissue sections were stained with a polyclonal antiserum against the HA of influenza B virus to assess the localization of virus spread. The images are representative of the clinical features of the tissue samples from each group, examined at the indicated magnifications. Extent of virus attachment and pathology were additionally noted along the maxillary surface of the nasal turbinates. Positive detection of viral antigen appears as brown staining. Magnification ×10 (*A*, *E*, *I*, *M*, *Q*, *U*), ×20 (*C*, *G*, *K*, *O*, *S*, *W*), ×40 (*B*, *F*, *J*, *N*, *R*, *V*), ×60 (*D*, *H*, *L*, *P*, *T*, *X*).

Only limited information is available about the immunohistopathologic characteristics of influenza B viruses in the ferret animal model, where severity of disease may be determined by the extent of involvement of the LRT tissues [39]. Therefore, we assessed the extent of viral spread in both the URT and LRT tissues collected at 3 dpi from ferrets inoculated with rg-WT or NAI-resistant influenza B viruses (Fig 2). In general, immunohistopathologic findings in all groups were restricted to the URT nasal mucosa, with both lesions and virus-positive cells being more abundant in the rostral (most anterior) sections of the respiratory mucosa; the extent and severity of virus infection was extremely limited in the caudal areas. The dorsal nasal turbinates were markedly thickened as a result of submucosal inflammation and edema, which were often accompanied by a moderate to marked nasal exudates and cellular debris plugging many nasal passages between turbinates (Fig 2A, 2B, 2I, 2J, 2Q, and 2R). In contrast to the URT, no noticeable lesions and essentially no evidence of virus infection were detected in the trachea and lungs in any group.

**Fig 3:**
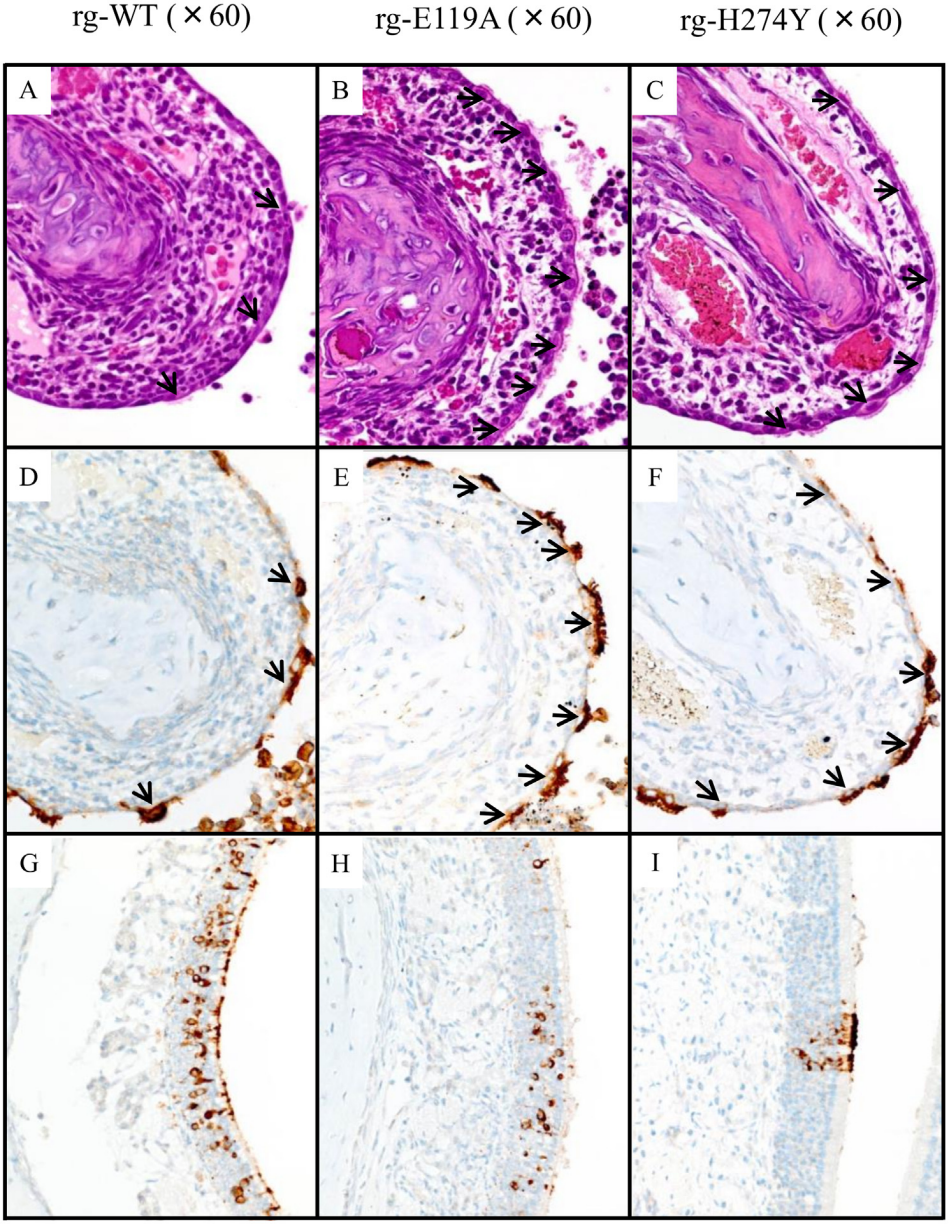
Differences in the thickening of the dorsal turbinates, attenuation of ciliated respiratory epithelium, and pattern of antigen detection in the olfactory epithelium in ferrets inoculated with rg-WT, rg-E119A, or rg-H274Y influenza B viruses. Widespread flattening and attenuation of the ciliated epithelium were observed in rg-WT–inoculated ferrets (A, D; arrows). In contrast, numerous ciliated cells remained in rg-E119A–inoculated ferrets (B, E), and intermediate numbers of ciliated cells survived in rg-H274Y–infected mucosa (C, F). In olfactory neurons, multiple variably sized patches of virus-positive cells were noted in rg-WT–inoculated ferrets (G) but were rare to absent and generally much smaller in animals inoculated with rg-E119A (H) or rg-H274Y (I). Positive detection of viral antigen appears as brown staining. Magnification ×60.

The loss of ciliated respiratory epithelium was most severe at the tips of the turbinates in all three groups; the flattened and attenuated epithelium in these areas was generally negative for virus antigen (Fig 2E, 2F, 2M, 2N, 2U, and 2V). Respiratory epithelial cells were intermittently positive in the rg-WT- and rg-H274Y–inoculated ferrets (Fig 2F and 2V), whereas they were more diffusely positive in the rg-E119A–inoculated ferrets (Fig 2N), a difference that most clearly evident in the middle and innermost regions of the trabeculae. At higher magnifications, widespread flattening and attenuation of the respiratory epithelium was evident in the rg-WT virus–inoculated group, with only a small number of ciliated epithelial cells remaining (Fig 3A and 3D; arrows). In contrast, numerous ciliated cells still lined the turbinate mucosa in animals inoculated with the rg-E119A virus (Fig 3B and 3E; arrows), whereas only an intermediate number of ciliated cells remained in the rg-H274Y virus–inoculated animals (Fig 3C and 3F; arrows). Multiple patches of virus-positive olfactory neurons were noted in the rg-WT–inoculated ferrets (Fig 3G) but were rare to absent and generally much smaller in the rg-E119A– and rg-H274Y–inoculated ferrets (Fig 3H and 3I).

Infection of the respiratory epithelium lining the maxillary surfaces was limited. Although no degeneration or necrosis was detected in HE-stained sections (Fig 2C, 2D, 2K, 2L, 2S, and 2T), IHC staining revealed small clusters of virus-positive cells (Fig 2G, 2H, 2O, 2P, 2W, and 2X) which were rare in the rg-E119A–inoculated ferrets (Fig 2O and 2P). Virus antigen was often restricted to the cilia (Fig 2P). In contrast, virus-positive cells in the rg-WT–and rg-H274Y–inoculated ferrets involved extensive areas (Fig 2G, 2H, 2W, and 2X) and was detected within the perinuclear cytoplasm indicative of virus replication underway (Fig 2H and 2X). Multiple variably sized patches of virus-positive olfactory neurons were noted in the rg-WT–inoculated ferrets (Fig 3J) but, they were rare to absent and generally much smaller in the rg-E119A– and rg-H274Y–inoculated ferrets (Fig 3K and 3L).

### Competitive fitness in ferrets, genetic stability, and acquisition of additional NA substitutions in the absence of antiviral drug pressure

**Fig 4:**
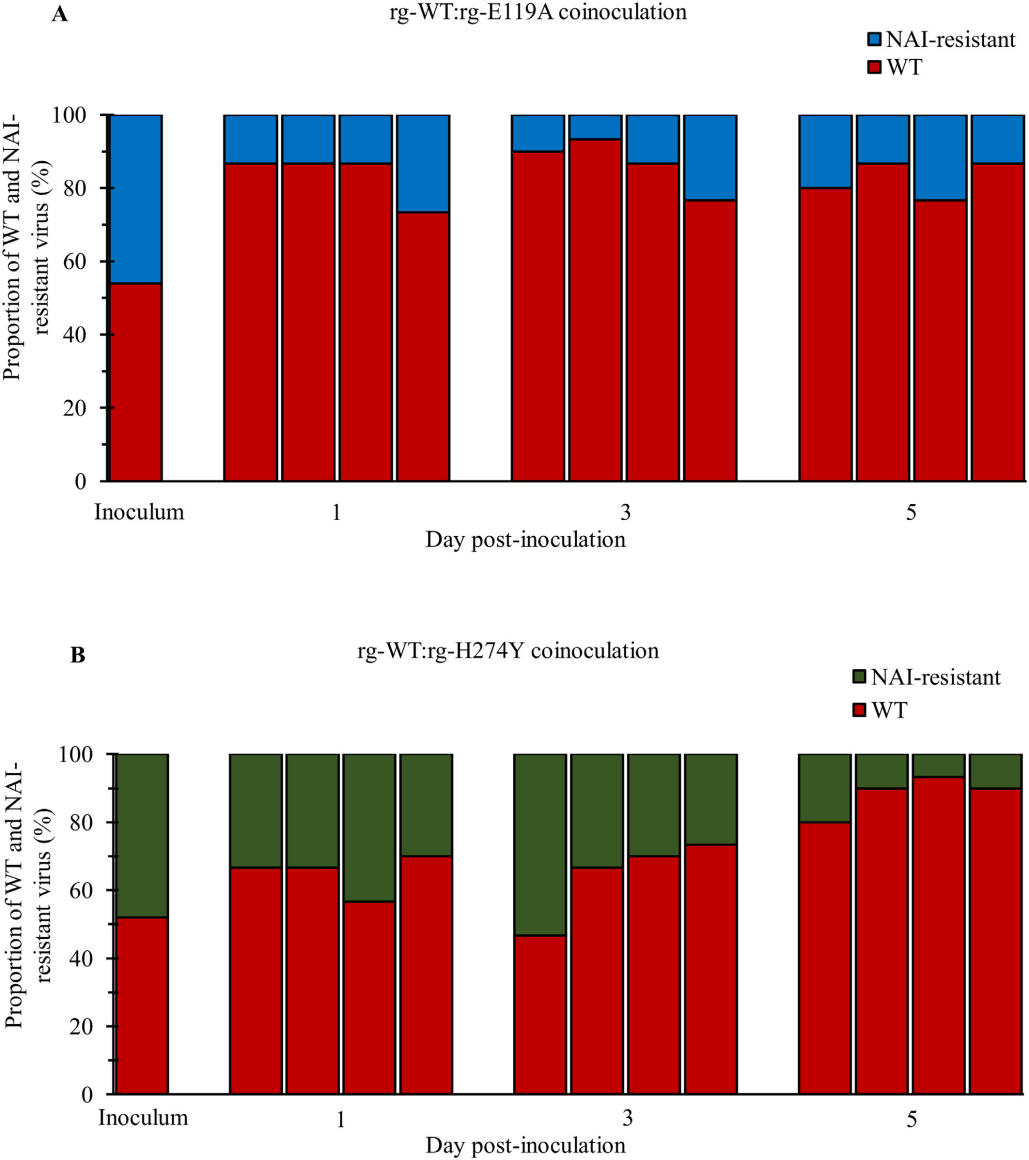
Relative virus proportions in the nasal washes of ferrets coinoculated with NAI-susceptible and -resistant influenza B viruses. The NA genes were amplified directly from nasal washes collected at 1, 3, 5, and 7 dpi from ferrets (*n* = 4/group) that were coinoculated intranasally with a mixture (1:1 ratio) of the NAI-susceptible rg-WT (5 × 10^5^ PFU) and NAI-resistant rg-E119A (A) or rg-H274Y (B) virus (5 × 10^5^ PFU) administered in 1 mL PBS. The proportions of rg-WT and NAI-resistant viruses were determined by TA clonal analysis and Sanger sequencing. At least 30 NA clones were analyzed for each animal; the results are expressed as a percentage of the total number of clones analyzed in each animal (*n* = 120 clones/group).

Coinoculation experiments, combined with genotypic analysis of virus populations, are helpful for examining virus-virus interactions within a host [40–42]. We assessed the predominance of NAI-susceptible versus NAI-resistant genotypes in virus populations isolated from the URTs of coinoculated ferrets (Fig 4). No changes in the NA sequence at position 119 or 274 were found in isolates from ferrets inoculated with either rg-E119A or rg-H274Y virus alone, indicating that E119A and H274Y NA substitutions in the B/Yamanashi/166/1998-like virus background were genetically stable in a ferret model without antiviral drug pressure. Accordingly, the rg-WT virus did not acquire either of these specific NA substitutions. In coinoculated ferrets, viruses recovered from nasal washes at 1, 3, and 5 dpi showed mixed genotypes in all animals, but rg-WT consistently represented the highest proportion of identified virus in the mixed virus populations. The proportion of rg-E119A in individual ferrets from the rg-WT:rg-E119A coinoculation group did not exceed 20% at any assessed time point, and the average proportion remained between 13.3% and 17.3% up to 5 dpi (Fig 4A). In contrast, the yield of rg-H274Y from the rg-WT:rg-H274Y coinoculation group represented up to 50% of the virus in one ferret at 3 dpi. However although the average proportion of rg-H274Y was 35% at 1 dpi, it gradually decreased to 20% over the course of infection (Fig 4B). Overall, the rg-WT virus maintained its predominance and superior fitness relative to the rg-E119A and rg-H274Y viruses in ferrets in the absence of drug pressure.

We also examined whether extra NA substitutions emerged during the course of virus replication in ferrets without the selection pressure from NAIs, particularly at the 19 conserved residues [12] and at supporting (i.e., not catalytic or framework) G109, E110, S250, T325, G402, and G142+146 residues, which have been associated with NAI resistance in influenza B viruses (Table 2) [9]. Furthermore, secondary permissive NA substitutions V233, V240, T288, D342, N368, and N385 were also targeted, because they individually enhance the fitness of NAI-resistant human influenza A (H1N1) viruses carrying H274Y substitution [43–46]. We did not find any of the permissive substitutions or changes at the supporting residues. However as listed in Table 2, we found substitutions at the catalytic and framework sites of NA which emerged sporadically without any antiviral drug selection pressure after replication in ferrets. These highly conserved positions among influenza A and B viruses have been previously proposed as candidate NAI resistance-associated sites [9,12,13]. Except for I222T, none of these NA substitutions (e.g., R118G, D151G, W178R, S179I, E227G, R371G, and E425K) have previously been detected among NAI-resistant influenza B viruses in surveillance or clinical settings [9,14]. However, all of the recorded NA substitutions in this study were at a low frequency (<1%), with only one or two being detected among the 120 clones randomly selected for sequence analysis (Table 2).

**Table 2:** Amino acid substitutions detected in the NA glycoproteins of NAI-susceptible and -resistant influenza B viruses recovered from nasal washes of inoculated and coinoculated ferrets.

Recombinant B/Yamanashi/166/1998 influenza virus	Ferret 1	Ferret 2	Ferret 3	Ferret 4
Virus-host interaction				
rg-WT	R118G^a^	-	-	-
	-	D151G^a^	-	-
rg-E119A	E425G^b^	-	-	-
rg-H274Y	-	R371G^a^	-	-
	-	-	-	I222T^b^
Virus-virus interactions within the host				
rg-WT:rg-E119A	-	S179I^b^	W178R^b^	-
rg-WT:rg-H274Y	-	E227G^b^	-	-

The NA genes were amplified directly from nasal washes collected at 5 dpi from ferrets (*n* = 4/group) inoculated intranasally with an individual virus (virus-host interaction group) or coinoculated with a mixture (1:1 ratio) of the NAI-susceptible rg-WT (5 × 10^5^ PFU) and an NAI-resistant virus (either rg-E119A or rg-H274Y, at 5 × 10^5^ PFU) administered in 1 mL PBS. The proportions of rg-WT and NAI-resistant viruses were determined by TA clonal analysis and Sanger sequencing. These NA substitutions were noted in just one of the 30 clones analyzed for each ferret (*n* = 120 clones/group). Dash lines indicate negative detection.

^a^Catalytic site NA residue.

^b^Framework site NA residue.

## Discussion

It is important to determine the replication and transmission fitness of NAI-resistant influenza B viruses to accurately evaluate the risks associated with the emergence and spread of antiviral drug-resistant viruses in the community [42,47–49]. Focusing on the replication fitness aspect, we studied here the effect of single naturally occurring NA substitutions (E119A and H274Y) in the homogeneous genetic background of the B/Yamanashi/166/1998-like virus (Yamagata lineage) in a ferret animal model. Virus fitness can be independently affected by differences in the virus genetic background and the location of the NAI resistance–associated NA substitution [15,16]. The use of viruses with identical genetic backbone allowed us to study the independent contribution of E119A and H274Y NA substitutions on replication fitness costs in a mammalian animal model. Our foregoing results showed that the two NA substitutions affected replication fitness in ferrets variably using single virus or mixed virus inoculation approaches. In traditional virus-host interaction experiments where ferrets were inoculated with a single virus, E119A demonstrated slightly diminished replication fitness relative to that of the parental rg-WT virus, whereas H274Y did not seem to perturb virus fitness. However, in a coinoculation ferret model, the NAI-resistant viruses could not outcompete and replace the rg-WT virus easily if at least equal proportions of the NAI-susceptible and -resistant viruses are present in the virus mixture. Thus, neither rg-E119A nor rg-H274Y has replication fitness advantage over rg-WT in direct competition experiments without antiviral drug pressure.

The presence of an E119A or H274Y NA substitution did not influence the mild clinical morbidity observed typically in ferrets experimentally inoculated with influenza B viruses [22–25,27]. Although both the 119 and 274 NA residues are framework sites that support the enzyme binding pocket [12], acquisition of E119A, but not H274Y, can potentially disrupt replication fitness in ferrets early during infection. However, rg-E119A was able to recuperate from the initial growth constraint. The E119A NA substitution has been shown to decrease NA enzyme activity while retaining substrate affinity (Km) and the rate of catalysis (Vmax) [18]. Hence, the impaired NA enzyme activity altered the virus replication. Meanwhile, the lack of readily observable defect in the growth characteristics of the rg-H274Y virus suggests that it may have relatively undiminished fitness as compared to rg-WT virus in ferrets inoculated with a single virus. It will be interesting to examine the effect of E119A and H274Y on the pathogenicity and fitness of influenza B viruses of Victoria lineage.

Histopathologic and IHC findings in URT tissues correlated well with the pattern of virus replication at the nasal cavity and the lack of detectable replication in the LRT tissues of ferrets. The presence of lesions and viral antigen predominantly in the anterior sections of the nasal turbinates suggests that B/Yamanashi/166/1998–like viruses preferentially replicate in ciliated cells of the URT which have been described to have higher capacity to produce and release human-origin influenza viruses, as compared to the alveolar cells of the LRT [50]. Moreover, these findings also suggest virus binding affinity to α2,6Gal-SA cell receptors that predominantly line the nasal mucosa of ferrets and preferential growth at low temperatures (≤33°C) [31,51,52] rather than conditions found in the LRT tissues (e.g., mixtures of α2,3Gal- and α2,6Gal-SA cell receptors and temperatures ≥ 37°C [39,53,54]. We also provide the first demonstration of the different immunohistopathologic features of NAI-susceptible and -resistant influenza B viruses in the URT of ferrets, at least for a Yamagata lineage virus. Interestingly, the rg-E119A virus demonstrated accumulation or clumping pattern of antigen detection along the ciliated respiratory epithelium compared to that caused by the rg-WT and rg-H274Y viruses. While this observation may seem to suggest to less sloughing off from infected ciliated cells, it may also correlate to altered NA enzyme activity of the rg-E119A virus as discussed above [18]. When combined with the virus replication data, these findings indicate that rg-H274Y has a certain degree of fitness advantage over rg-E119A. Altogether, the subclinical influenza B virus infection in ferrets can be attributed to (1) moderate virus replication at the URT; (2) the absence of severe lesions or evidence of virus replication in the LRT; and (3) the lack of pronounced changes in laboratory indicators of inflammation orchestrated at the site of active virus replication. Surprisingly, despite generally similar URT viral titers, the NAI-resistant viruses (both individually and in 1:1 coinoculation with rg-WT) appeared to induce more inflammatory response than did rg-WT virus albeit were not always significant. It is possible to suggest that the presence of defective interfering particles in the stocks of NAI-resistant viruses could cause stronger recruitment of inflammatory cell population [55,56]. However, how these noninfectious but biologically active subpopulations exactly affect or induce different biological properties (e.g., activation of innate immune response) remains to be studied.

There is only limited information on the fitness of NAI-resistant influenza B viruses in competitive coinoculation ferret model. It has been shown that the catalytic R152K NA substitution diminished the fitness of B/Memphis/20/1996 (Yamagata lineage) virus in coinoculated ferrets without antiviral drug administration; treatment of ferrets with zanamivir reverses the relative fitness of the mutant virus [27]. In contrast, the framework D198N NA substitution did not significantly affect competitive fitness of B/Rochester/02/2001 virus in the presence or absence of drug pressure [28]. Competitive viral fitness, as based on the development of clinical signs of infection and virus replication kinetics, was not evident among ferrets inoculated with the rg-WT:rg-E119A or rg-WT:rg-H274Y mixture. However, genotypic analysis revealed that NAI-resistant variants could not efficiently outcompete or displace the NAI-susceptible virus indicating that the rg-WT virus would then still be selected in the virus population during mixed virus infections. Since this study was performed in the absence of antiviral drug pressure, it will be necessary to determine whether rg-E119A or rg-H274Y viruses would gain a replicative fitness advantage in NAI-treated animals through the inhibition of NAI-susceptible variants that compete for dominance in the infected host.

The fitness of NAI-resistant viruses can be improved by the acquisition of additional viral genome modifications including, but not limited to, the NA gene segment. In this study, secondary permissive NA substitutions previously linked to H274Y [43–46] or modifications at residues supporting the NA active site [9] were not observed in viruses recovered from the inoculated ferrets. Although NA substitutions at the catalytic (e.g., R118G, D151G, R371G) and framework (e.g., W178R, S179I, I222T, E227G, E425G) sites were found after replication in the ferret hosts, it is important to note that only I222T have been detected among NAI-resistant influenza B viruses in clinical and surveillance settings [9]. All NA substitutions were determined at low frequency which can be an indicator of diminished virus fitness. Alternatively, these substitutions can be easily masked by the dominant strain in a heterogenous virus population and can be overlooked by commonly used methods of genetic identification, including direct Sanger sequencing of clinical samples. While some of these NA substitutions may be host-adaptive in nature, their association with antiviral drug resistance in the context of influenza B virus background warrants further investigation. Additionally, their potential role as permissive or compensatory substitutions to restore possible virus fitness defects arising from the E119A or H274Y NA protein modification is not completely ruled out. Notably, optimal balance between the receptor-binding affinity of the HA protein and the virus-releasing activity of the NA protein is critical for influenza virus replication [57–59].

With the increasing use and stockpiling of NAIs, which remain the only class of antiviral drug recommended for treating influenza B virus infections, it is important to further investigate the emergence of NAI-resistant viruses that retain replication fitness in humans. Crucial addenda to our line of investigation would be the analysis of mixed virus populations in ferrets undergoing NAI treatment and the transmission fitness of NAI-resistant influenza B viruses in this host which will decisively determine overall viral fitness. Altogether, our findings indicate that the order of relative fitness of the influenza B viruses in a ferret animal model studied is: rg-WT > rg-H274Y > rg-E119A. Although this order seems to differ to their proposed relative fitness in cell culture model [29], it should be noted that *in vitro* studies may not necessarily reflect replication fitness in animal models due to limited host antiviral mechanisms present in cell lines [60,61]. Hence, the current study highlights the need for corresponding investigations using *in vivo* model system to accompany findings *in vitro*. Given that the NAI-resistant viruses investigated here, particularly rg-H274Y, demonstrated replicative ability in ferrets despite their inability to outcompete the NAI-susceptible virus counterpart, our findings still underscore the need to monitor the emergence of these and other viruses bearing NAI resistance–associated NA substitutions. Furthermore, these results emphasize the continuous risk assessment of potential drug-resistant influenza B viruses; this will be beneficial for optimizing antiviral drug treatment, as the clinical consequences of NAI resistance–related substitutions remain uncertain and may limit options for treatment.
